# Focused Research on the Challenges and Productivity of Researchers in Nigerian Academic Institutions Without Funding

**DOI:** 10.3389/frma.2021.727228

**Published:** 2021-10-28

**Authors:** Bernard E. Igiri, Stanley I. R. Okoduwa, Ebere P. Akabuogu, Ugochi J. Okoduwa, Idongesit A. Enang, Olanipekun O. Idowu, Suleiman Abdullahi, Imeh E. Onukak, Catherine C. Onuruka, Ogechukwu P.O. Christopher, Akinbobola O. Salawu, Aimee O. Chris, David I. Onyemachi

**Affiliations:** ^1^ Directorate of Research and Development, Nigerian Institute of Leather and Science Technology (NILEST), Zaria, Nigeria; ^2^ Department of Biochemistry, Babcock University, Ilishan-Remo, Nigeria; ^3^ Industrial and Environmental Pollution Department, National Research Institute for Chemical Technology, (NARICT), Zaria, Nigeria; ^4^ Scientific and Industrial Research Department, NARICT, Zaria, Nigeria; ^5^ North Central Regional Extension Centre, NILEST, Utako-Abuja, Nigeria; ^6^ South-East Extension Services Centre, NILEST, Owerri, Nigeria; ^7^ South-West Liaison Office, NILEST, Ilara-Remo, Nigeria

**Keywords:** productivity, challenges, publication, academic staff, career progression, researchers, research policy

## Abstract

**Background:** The challenge of research funding constraints has brought to bear enormous pressure on researchers. Research productivity is relevant to prestige and career progression of academic staff. However, this study aimed to explore significant challenges associated with researchers’ productivity and the impact of non-funding of research in Nigerian research and tertiary institutions.

**Methods:** This study adopted a qualitative exploratory design involving academics at various research and tertiary institutions across the six geographical regions in Nigeria. A semi-structured questionnaire was distributed electronically to all participants who consented to take part in this study. Exactly 4,159 questionnaires were administered and 2,350 were completely filled and returned. Pearson correlation matrices with logistic regression were used for data analysis and are presented in frequencies and percentages.

**Results:** On challenges faced by respondents, 42.98% reported a lack of research funding, 17.11% mentioned brain drain challenge while 8.85% indicated a lack of motivation. Of the 23,927 publications reported, the number of those in sciences, engineering, and medical sciences averaged 9.6, 11.5, and 9.5 respectively. The average number of publications by women (10.8) was more than by men (9.7). Lecturers had the highest average research publication number (11.8) followed by researchers (10.2) and others (3.9). Men had the highest (11.9) average number of conferences compared to women (9.2). Participants in engineering had an average number of 13.8 conferences per respondents followed by those in education (11.2), sciences (11.1), and 10.9 for those in agricultural sciences. The result revealed a negative significant correlation between research publication and academic qualification at *p* < 0.01. Positive significant correlation was observed between research productivity and discipline at *p* < 0.05. Findings show that the combined influence of the independent variables on research productivity was significant using linear regression analysis.

**Conclusions:** The failure to prioritize research has resulted in underdevelopment in Nigeria. It is therefore imperative that the federal government prioritize research and establish a functional Special Research Trust Fund to oversee research funding in Nigeria.

## Introduction

Research includes scientific investigations conducted to explore new facts, and its activities are significant in propelling the developmental process of any nation. The primary function of research is to explore answers to meaningful questions aimed at improving societal challenges (Lucky 2013)*.* There is a relationship between research and development (R&D) in comparison to productivity, of which both play essential roles in the economic growth of the nation and building of human capacity (Bayarçelik and Taşel, 2012; Blanco et al., 2016). In the quest to advance knowledge, researcher’s performance is evaluated based on research outcomes in terms of productivity (Appah et al., 2020), which could be used by other scholars, stakeholders, policymakers, industries, and the wider society. In academia, research productivity is the measure of publication counts of articles published in “peer-reviewed” journals, referred books, book chapters, *h*-index, awarded research grants, conference proceedings, and patents of academics (Butler, 2003; Ball 2005; Hirsch, 2005; Beerkens 2013; Moeheriono 2014; Akbaritabar et al., 2018; Oyeyemi et al., 2019).

Research productivity increases the social prestige of an academic staff member and it is associated with appointments, promotion, and high salaries (Federal Republic of Nigeria 2000; Kotrlik et al., 2002; Bassey et al., 2007; Chiemeke et al., 2009). Researchers with high reputation status are likely to publish in journals with high impact factors when compared to researchers whose reputation are ranked among those of low status. Bibliometric methods such as *h-*index, citation counts, and publication counts are used to ascertain the impact of an article among scholars (Carpenter et al., 2014; Penner et al., 2013; Dolenc et al., 2016; Agarwal et al., 2016; Moher et al., 2018). Research funding is low in most Africa countries (Saric et al., 2018). A universal evaluation of R&D as a fraction of Gross Development Product (GDP) reveals that many African countries spend less than 1% on research and development (Karimi, 2015; Maiyo, 2015; UNESCO Institute of Statistics, 2018). Ebikabowei et al. (2017) reported that a greater number of research studies carried out by academic staff in Nigeria are self-funded from their inadequate salary. Although, in Nigeria, the Tertiary Education Trust Fund (TETFund) is the highest funding body that funds research in tertiary institutions (Universities, Polytechnics, and Colleges of Education) it however excludes research institutes (whose sole mandate is focused on research) from benefitting from research funding. Policymaking in the selection of research proposals, inadequate publicity for research grants applications, and lack of knowledge about funding agencies are the major hindrances to accessing research grants in Nigeria**.**

Several government research institutes and universities in Nigeria conduct research, but these institutions often face serious challenges, particularly gross underfunding and disconnect from national research priorities (Federal Ministry of Health Nigeria, 2018), which significantly affect the output of their mandates and national development. The absence of core funding for research-focused institutes has been recognized as a major constraint to the development of Africa (Shroff et al., 2017; Grepin et al., 2017). However, in many African countries including Nigeria, the research sector faces the toughest challenges (Njuguna and Itegi, 2013). Nigeria has numerous research institutions, universities, polytechnics, and monotechnics that are owned by the federal, state, and private sectors (Supplementary Material S1), yet, are confronted with a series of challenges and brain drain. The greatest challenges affecting Nigerian researchers from development and survival in order to meet sustainable development goals (SDGs) are family challenges, financial constraints, inadequate research skills, inadequate motivation from employer, brain drain, inadequate training, too many administrative duties, inadequate mentoring, heavy workload (leaving little time for research), inadequate research grants, infrastructural inadequacy, research misconduct, lack of research funding, and inadequate information resources in the library (Chikwe et al., 2015; Kumwenda 2017 **;** Emakoji and Otah 2018; Okoduwa et al., 2018; Fayomi et al., 2019; Ezeanolue et al., 2019). The research climate is an important factor that encourages integrity in research. However, little is known about what constitutes a responsible research climate (Bouter 2015: Haven et al., 2020). There are challenges associated with the working culture in research with significant impact on researchers’ performance (Woolston, 2018; Van Noorden, 2008; The Nuffield Council on Bioethics, 2014; The Royal Society, 2017). The recent focal point on the measures of performance and productivity significantly poses challenges to researchers, research quality, and the relationship of research with society (Jones and Wilsdon, 2018; The Nuffield Council on Bioethics, 2014; The Royal Society, 2017). The development of skilled manpower associated with research productivity would advance the national economy of the country and significantly benefit the competitive globalized economy of the changing Information Communication and Technology Age (Kpolovie and Lale, 2017).

Various studies have ascertained factors that influence research productivity of academics (Teodorescu, 2000; Bland et al., 2005; Quimbo and Salabu, 2014; Kwiek, 2016; Negash et al., 2019). These factors have been categorized into individual and environmental factors (Teodorescu, 2000; Bland et al., 2005; Shin and Cumming, 2010). The individual factors include motivation, gender, age, research knowledge and skills, academic rank, research orientation, and collaboration while the environmental factors are leadership, availability of resources, institutional missions, orientation, rewards, mentoring programs, and institutional research policy. Nigeria research and tertiary institutions have experienced serious hindrances in research activities (Okoduwa et al., 2018). It is worrisome to note that Nigerian research institutes and universities are fast decaying. The resources required for research activities are all seriously inadequate and in bad shape to carry out the basic function. The order of research performance creates a working way of life that places more value on what is achieved and less on how it is achieved and the human expenses associated with it (Chubb and Watermeyer, 2017; Moore et al., 2017; Woolston 2018, Van Noorden 2008, Woolston 2019; Parr, 2015; The Nuffield Council on Bioethics, 2014; The Royal Society, 2017; Wilsdon, 2016). The deteriorating research quality in Nigeria and on the African continent is attributed to the moribund investment in research (Olukoju 2002). By 2030, the Sustainable Development Goals are to promote research in all fields and for full research capacity in all countries of the world (UN 2015). The future of research in Africa is in the hands of its young scientists, but there is little or nothing done to support them (Friesenhahn and Beaudry 2014). The circumstances stated above propose that African countries, particularly Nigeria must reconsider their research agenda taking cognizance of the essential role played by research in the development agenda (Mwendera et al., 2017). In light of the above shortcoming, the research institutes and universities in Nigeria lack the research capacity which, according to UNESCO (2006) is the aggregate of human, institutional, and financial conditions for pursuing research. It is against this backdrop and the recognition of this limitation, that this study embarks on the impact of the challenges and productivity of researchers that lack research funding with emphasis on Nigeria in order to ascertain their effort in contribution to the development of the nation’s economy through their scientific work.

## Research Methods

### Study Design

This study adopted a qualitative exploratory design as a research methodology and a semi-structured questionnaire approach, which was distributed to academic staff of research institutes, universities, and polytechnics across the different geographical regions of Nigeria. The comprehensive details about the various research and academic institutions in Nigeria are presented in the Supplementary Material section. The questionnaire was distributed to academic staff in different disciplines such as sciences, medical sciences, social sciences, education, engineering, and humanities.

### Sampling

The respondents in this study comprise all cadres of researchers, lecturers, and others that are also involved in research activities, which constitute the academic staff of the institutions. The survey questionnaire was administered and delivered to participants through WhatsApp, emails, Facebook, and Telegram. A reminder was sent to the participant after the return deadline for those that did not respond. It took about 5–10 min to complete the questionnaire.

### Study Procedures

The questionnaire was subdivided into various thematic sections. Thematic A of this questionnaire requested information on socio-demographic variables such as gender, age, geographical area, designation, discipline, highest qualification, and the name of organization. Thematic B requested information on variables relating to researchers’ productivities in terms of number of peer-reviewed articles, amount of conferences, citation counts, *h*-index, and publication prestige. Thematic C asked for information about researchers’ challenges with respect to factors that limit research performance, factors that motivate effective research productivity, institutional challenges, career progression, and perception about research. The questionnaire was pretested and distributed to various academic staff of research institutes, universities, and polytechnics that were representative of all regions (Supplementary Figures S1, S2 and Supplementary Table S1–Supplementary Table S14). The study location was stratified into six regions, north west, south west, south-south, south east, north central, and north east. Eleven institutions (name withheld for ethical reasons) were selected from each zone using a simple random method. In all, 66 institutions (33 universities, 18 research institutes, and 15 polytechnics) were included in the study where a disproportionate quota-sampling method was used to select participants from each institution within a region totaling 2,350 participants (1,087 lecturers, 982 researchers, and 285 others) from the six regions. The information (such as publications and citation data, etc.) provided by the respondents was randomly checked through Google Scholar for the purpose of verification and certification of accuracy. The study was conducted between December 5, 2020 and August 15, 2021.

### Selection Criteria

The participants in this study comprised all cadres of researchers, lecturers, technologists, and instructors constituting the academic staff of research institutes, universities, and polytechnics in Nigeria. Non-research and administrative staff were excluded from the study.

### Grouping

For the purpose of this study, all academic staff (from the universities, research institutions, and polytechnics) involved directly in teaching were grouped as lecturers. Staff involved strictly in research focused activities were grouped as researchers. Other academic staff involved in laboratory practicals such as technologists and instructors was grouped as others.

### Statistical Analysis

Data on publications, conference papers, *h*-index, and citation counts were obtained from the returned questionnaire. Completed questionnaires were received via Google form, coded, entered, and analyzed using the Statistical Package for the Social Science (SPSS), version 25.0 for Windows (SPSS Inc., Chicago, Illinois, United States). The analytical approaches used were descriptive statistics such as percentages and frequencies. The relationships between the different variables were assessed by Pearson’s rank correlation. Data were expressed in mean and standard deviation and were compared using analysis of variance (ANOVA). *p* < 0.05 was considered statistically significant.

## Results

### Baseline Demographic Characteristics of the Studied Participants

**FIGURE 1:**
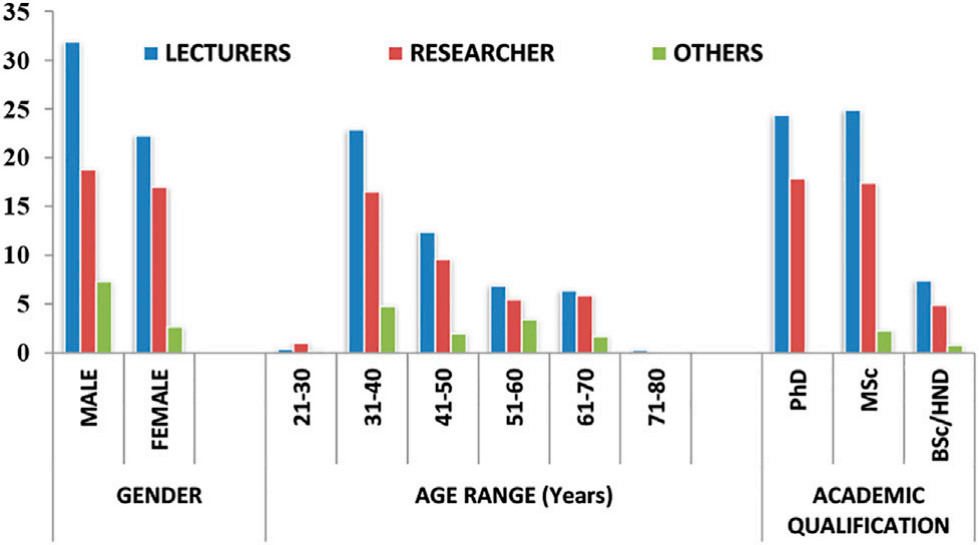
Demographic characteristics of respondents in percentages.

The percentage distribution of respondents and their demographic characteristics is presented in Figure 1. A total of 4,159 questionnaires were distributed, of which 2,350 respondents completed and returned the questionnaire giving a response rate of 56.5%. More male (*n* = 1,363, 58%) than females (*n* = 986, 42%) academics participated in the study. Of this proportion, 31.9% were male lecturers; 22.3% female lecturers, 18.8% male researchers, 17.0% female researchers, 7.3% male others and 2.7% female others (Figure 1). There was no significant statistical association between gender and research productivity at *p >* 0.05 (Table). The majority of the respondents (40.3%) were within the age range of 31–40 years. Among this, 22.9% were lecturers, 16.5% researchers, and 4.8% others. Exactly 44.6% indicated an MSc degree as their highest educational qualification, with 24.9% lecturers, 17.4% researchers, and 2.3% others (Figure 1). A total of 42.3% of respondents indicated PhD, while 13.1% indicated Bachelor of Science Degree/Higher National Diploma (BSc/HND) as their highest qualification.

The participants were drawn from Nigeria universities, research institutes, and polytechnics with 56.6, 30.5 and 12.9% of respondents, respectively (Table 1). A total of 35.2% of the study participants specified that their institution is situated in the north west, 15.3% south-south, 11.9% north central, 19.5% south west, 12.1% south east, and 6.0% north east.

**TABLE 1:** Percentage distribution of respondents by institutions and designations.

Institutions	Researcher	Lecturer	Other	Total
University (%)	408 (17.4)	795 (33.8)	127 (5.4)	1,330 (56.6)
Research institute (%)	515 (21.9)	96 (4.1)	105 (4.5)	716 (30.5)
Polytechnic (%)	59 (2.5)	192 (8.2)	53 (2.3)	304 (12.9)
Total	982 (41.8)	1,083 (46.1)	285 (12.1)	2350 (100)

**FIGURE 2:**
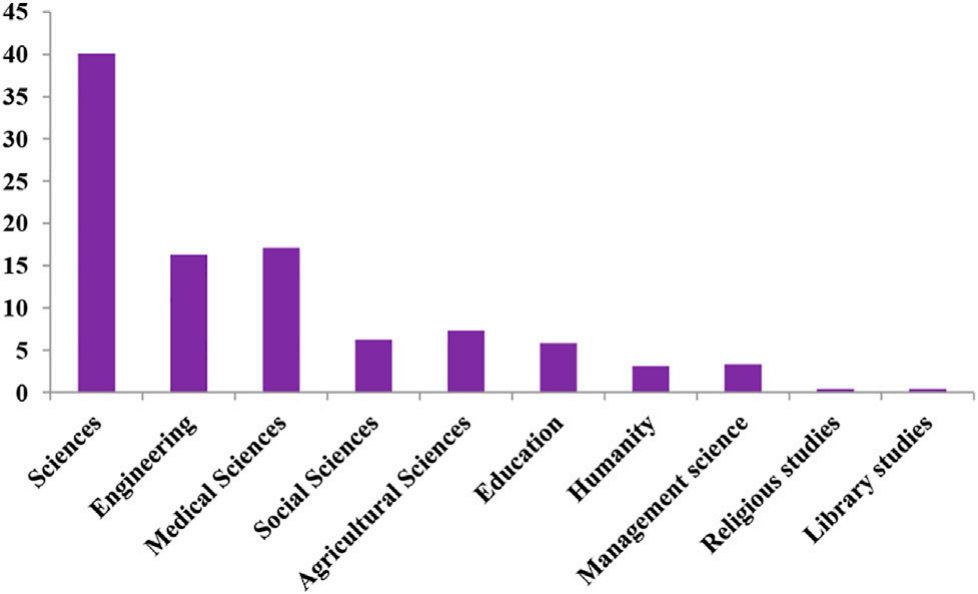
Percentage of respondents with respect to discipline.

The majority (44.7%) of the participants were in the sciences, while 16.3% were in engineering, 17.5% medical sciences, 5.7% in social sciences, 2.3% humanities, and 5.6% in agricultural science (Figure 2). Overall, 70.3% of the respondents were full-time staff, 27.5% on sabbatical/secondment, and 2.2% were adjunct staff. Based on the designations of the participants, the lecturing cadre had the highest number with 46.1% of respondents, while 41.8% were researchers and 12.1% others.

On participants’ career progression, 40.2% of respondents strongly agree that they feel satisfied with their career prospect within research while 28.9% agree, 20.3% disagree, and 10.6% strongly disagree. In total, 35.9% of respondents agree that they feel satisfied if a research professor is awarded in research institutes, while 29.5% strongly agree, 21.6% were neutral, 8.5% disagree, and 4.5% strongly disagree.

### Participants Performance in Terms of Productivity

The total number of publications reported by the participants was 23,927. Of this number, men had average publications of 9.9 per male respondent while women had an average publication of 10.8 per female respondent. The highest average research publications (11.6 and 10.8) were recorded by those in age group 61–70 and 51–60 years (Table 2). However, those within the age group 71–80 had the highest average citation count (28.4) followed by those of 41–50 years (24.1) (Table 2). The least average citation count (2.6) was recorded among those of 20–30 years. The academics in education had an average research publication number of 13.8, those in management sciences had 12.7, engineering had 11.5, religious studies 10.9, social sciences 9.9, and sciences 9.6 (Table 2). Those with PhDs had an average research publication number of 11.1 per respondents followed by MSc (10.4) and HND/BSc (6.3) achievers. The lecturers had the highest (11.8) average research publication number followed by researchers (10.2) and others (3.9). With respect to the average number of conference papers, women had 13.5 while men had 13.2 average citation counts per respondents. The engineering specialty had 21.2, religious studies had 18.4, medical sciences had 17.7, and sciences had 11.6 average citation counts per respondents. Men had a higher (11.9) average number of conferences than women (9.2). Participants in engineering had an average number of 13.8 conferences per respondents followed by those in education (11.2), sciences (11.1), and 10.9 for those in agricultural sciences (Table 2). Those with PhDs had the highest (13.1) average number of conferences per respondent followed by those with MSc (10.7), and the list was recorded in those with HND/BSc. The lecturers had the highest (12.6) average number of conferences per participants followed by researchers (11.5) and others (1.4). The findings interestingly revealed a negative significant correlation between research publication and academic qualifications (r =−0.131**, *p* = (0.000) < 0.01) and between research publication and designations (r =−0.114**, *p* = (0.000) < 0.01) (Table 3). The study also established a positive significant correlation between research publication and age (r = 0.063**, *p* = (0.002) < 0.01) and between research publication and discipline (r = 0.048*, *p* = 0.021) < 0.05) (Table 3). Findings showed a positive statistically significant correlation between research productivity and gender (r = 0 0.052*, *p* = (0.012) < 0.05) (Table 3). The respondents’ productivity output shows a mean and standard deviation of *X* = 10.2, S.D. = 10.195 (Table 3). On the frequency of publication output, the majority (52.1%) reported that they publish at least one article in 2 years, 18.6% publish at least one article per year, 12.8% publish at least two articles per year, 16.5% publish at least four articles per year.

**TABLE 2:** Average research productivity of academics that participated in the study.

Participants bio data	Research publications	Citation counts	Conferences	*h-*index	*p* value
Gender					
Male	9.7	13.2	11.9	2.9	0.304
Female	10.8	13.5	9.2	2.3	
Age (years)					
20–30	8.4	2.6	9.8	1.4	0.001
31–40	9.8	10.3	10.9	2.5	
41–50	9.8	24.1	12.8	2.9	
51–60	10.8	14.6	11.4	2.7	
61–70	11.6	3.9	6.1	2.5	
71–80	9.6	28.4	15.4	11.3	
Discipline					
Sciences	9.6	11.6	11.1	1.7	0.00
Medical science	9.5	17.7	9.0	2.7	
Engineering	11.5	21.2	13.8	2.9	
Education	13.8	5.7	11.2	3.8	
Social sciences	9.9	6.9	7.4	5.3	
Humanities	9.7	5.7	6.6	1.0	
Agric. sciences	8.8	11.5	10.9	5.2	
Management sci	12.7	9.5	10.9	1.5	
Religious studies	10.9	18.4	9.3	2.5	
Library studies	5.2	9.9	6.6	1.2	
Qualification					
PhD	11.1	15.8	13.1	3.2	0.025
MSc	10.4	13.3	10.7	2.2	
HND/BSc	6.3	5.4	3.6	2.6	
Designation					
Lecturers	11.8	14.9	12.6	3.3	0.014
Researchers	10.2	14.2	11.5	2.4	
Others	3.9	4.3	1.4	1.1	
Challenges					
Non-funding of research	12.5	7.1	9.9	1.2	0.000
Brain drain challenge	10.4	35.3	18.0	2.9	
Lack of motivation	13.1	19.0	7.5	4.4	
Non-payment of hazards	7.6	25.6	10.4	5.5	
Lack of facilitating platform	8.0	10.7	10.6	5.8	
Stiff career pathway	4.7	9.1	3.8	2.1	
Lack of expertise	2.9	6.8	17.8	4.8	
Technical challenges	3.0	0.9	4.4	1.0	
Lack of research and writing skills	5.8	0.4	7.2	2.1	
Lack of mentorship	15.7	2.6	13.9	7.8	
Other challenges	2.2	0.0	2.9	2.3	

**TABLE 3:** Descriptive statistic and correlations matrix between demographic factors and research publication of respondents.

S/N	Variable	Mean	S.D.	1	2	3	4	5	6	7
**1**	RP	10.2	10.195	1						
2	GE	1.24	0.494	0.052* 0.012	1					
**3**	AG	2.98	1.12	0.063** 0.002	−0.011 0.610	1				
**4**	DI	3.31	2.444	0.048* 0.021	−0.014 0.494	0.060** 0.004	1			
**5**	AQ	1.71	0.685	−0.131** 0.000	−015 0.479	−0.093** 0.000	−0.068** 0.001	1		
**6**	DE	1.7	0.672	−0.114** 0.000	−0.007 0.730	−0.043* 0.039	−0.034 0.096	0.155** 0.000	1	
**7**	CH	14.1	9.184	0.150** 0.000	0.087** 0.000	0.110** 0.000	0.060** 0.003	0.152** 0.000	−0.042* 0.042	1

**Sig. at 0.01 level, * sig. at 0.05 level (two-tailed).

RP: Research publication, GE: Gender, AG: Age, DI: Discipline, AQ: Academic qualification, DE: Designation, CH: Challenges.

Table 4 shows the combined contribution of the independent variables (gender, age, discipline, academic qualification, designation, and challenges) to the prediction of research productivity using linear regression analysis. The result shows a coefficient of multiple correlations (r = 0.237, R2 = 0.056). This implies that 5.6% of the variance is accounted for by the predictor variables when taken together. The significance of the combined contribution was tested at *p* < 0.05. The result also revealed that the analysis of variance (ANOVA) for the regression yielded an F-ratio of 23.186 at a significance level of 0.05. This shows that the combined influence of the independent variables to the research productivity was significant (Table 4).

**TABLE 4:** The combined contribution of the demographic factors (gender, age, discipline, academic qualification, designation, and challenges) on research productivity.

R	R Square		Adjusted R square		Std. error of the estimate	
**0.237**	**0.056**		**0.054**		**9.918**	
**ANOVA**						
**Model**	**Sum of squares**	**DF**	**Mean square**	**F**	**Sig**	**Remark**
Regression	13,683.604	6	2280.601	23.186	0.000	Significant
Residual	230,459.809	2,343	98.361			
Total	244,143.413	2,349				

### Challenges Faced by Researchers in Their Career Progression

On the basis of challenges faced by researchers, 42.98% of the respondents indicated lack of research funding, 17.11% consented to the brain drain challenge while 8.85% reported a lack of motivation and 5.4% were of the view that non-payment of hazard and publication allowances are major challenges facing research and research activities in Nigeria academic institutions (Table 5). There is a positive significant correlation between research publication and challenges (r = 0.150**, *p =* (0.000) < 0.01) (Table 3).

**TABLE 5:** Challenges faced by researchers in Nigeria institutions.

Category	Participants	Frequency	Percentage (%)
Non-funding of research (42.98%)	Lecturers	644	27.4
	Researchers	317	13.5
	Others	49	2.1
Brain drain challenge (17.11%)	Lecturers	219	9.3
	Researchers	168	7.1
	Others	15	0.6
Lack of motivation (8.85%)	Lecturers	96	4.1
	Researchers	102	4.3
	Others	10	0.4
Non-payment of hazard and publication allowances (5.40%)	Lecturers	74	3.1
	Researchers	37	1.6
	Others	16	0.7
Lack of facilitating platform (4.38%)	Lecturers	61	2.6
	Researchers	32	1.4
	Others	10	0.4
Stiff career pathway (3.49%)	Lecturers	39	1.7
	Researchers	35	1.5
	Others	8	0.3
Lack of expertise (4.13%)	Lecturers	36	1.5
	Researchers	58	2.5
	Others	3	0.1
Technical challenges (4.68%)	Lecturers	69	2.9
	Researchers	33	1.4
	Others	8	0.3
Lack of research and writing skills (4.04%)	Lecturers	37	1.6
	Researchers	52	2.2
	Others	6	0.3
Lack of mentorship (4.94%)	Lecturers	64	2.7
	Researchers	43	1.8
	Others	9	0.4

On factors that promote and motivate effective research performance (Table 6), 68.5% of the respondents reported that adequate funding of research will motivate effective research performance, while 37.4% were of the view that collaborative research is a practice that will enhance research outputs. Overall, 36.3% of the respondents revealed that their career will be promoted through their aspiration to attain academic qualification while 51.1% responded that lack of research funding is a major factor that limits research productivity (Table 6).

**TABLE 6:** Factors that promote and enhance effective research performance.

Career promoting factors	Frequency (%)	Percentage
Aspiration to attain academic qualification	854	36.3
Work satisfaction	1,032	43.9
Money	145	6.2
None of the above	153	6.5
Formal classroom learning	92	3.9
Desire to fulfil parents dreams	74	3.1
Factors that limit researchers’ performance		
Lack of research funding	1,202	51.1
Lack of training	498	21.2
Lack of mentorship	224	9.5
Institutional climate	153	6.5
Age	85	3.6
Academic qualification	115	4.9
Lack of competence	49	2.1
Others	24	1.0
Factors that motivate researchers’ performan**ce**		
Adequate funding of research	1,610	68.5
Modern research facilities, equipment, and infrastructures	261	11.1
Putting in place appropriate reward structures	349	14.9
Payment of hazard and publication allowances	130	5.5
Practices that enhance research performance		
Collaborative research	879	37.4
Multi-disciplinary research	574	24.4
Conference and journal publication	792	33.7
Metricization	95	4.0
h-index evaluation	10	0.4

### Perception of participants about research and research activities

The perception of participants to research and research activities is presented in Table 7. From the perspective of the different groups of respondents, 37.4% indicated that research is good, but policymakers do not prioritize research. Among these groups, 25.4% were lecturers, 10.5% researchers, and 1.4% others. Overall, 28.2% of respondents indicated that the functionality of research determines the development of any nation. Among these categories of respondents 18.0% were lecturers, 9.1% research fellows, and 1.1% others. In total, 11.3% of the participants were of the view that research is demanding and impoverishes researchers due to non-funding of research. In these category of respondents, 5.6% were lecturers, 4.1% research fellows, and 1.6% others. A total of 12.9% of the respondents agreed that the progress of a nation is a function of its research administrative effectiveness. Among this group, 4.6% were lecturers, 7.6% research fellows, and 0.6% others (Table 7).

**TABLE 7:** Perception of respondents with respect to research and research activities.

Category	Participants	Frequency	Percentage (%)
The functionality of research determines development (31.4%)	Lecturers	423	18
	Researchers	213	9.1
	Others	26	1.1
Progress of a nation is a function of its research administrative effectiveness (17.0%)	Lecturers	108	4.6
	Researchers	179	7.6
	Others	15	0.6
I dislike research because of the technicality involved (1.7%)	Lecturers	44	1.9
	Researchers	98	4.2
	Others	15	0.6
Research is good, but policymakers do not prioritize research (32.0%)	Lecturers	598	25.4
	Researchers	247	10.5
	Others	34	1.4
Research is demanding and impoverishes researchers due to non-funding (17.3%)	Lecturers	131	5.6
	Researchers	96	4.1
	Others	37	1.6
Other (specify) (0.6%)	Lecturers	43	1.8
	Researchers	29	1.2
	Others	13	0.6

**FIGURE 3:**
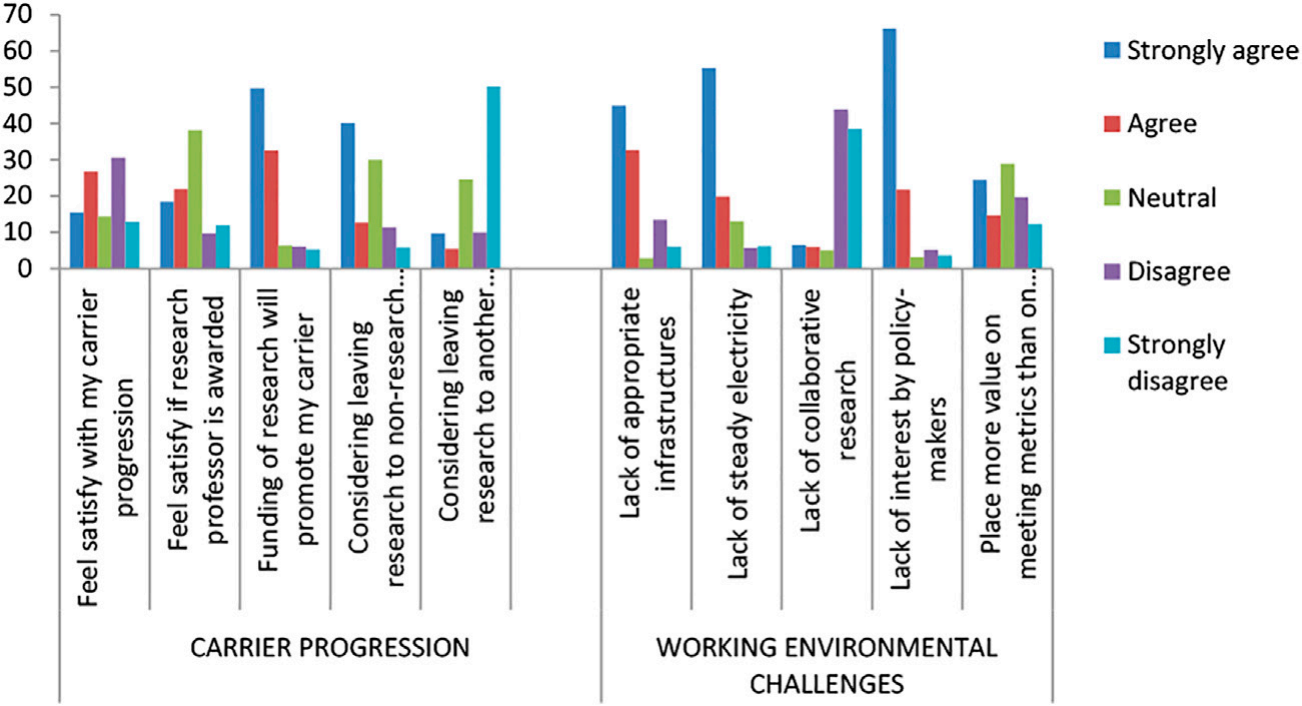
Participant responses with respect to career progression and working environmental challenges.

On participant’s career progression and working environmental challenges (Figure 3), 15.5% strongly agree that they feel satisfied with their career prospects working within the research environment. Exactly 21.9% agree that they will be fully satisfied if a research professor is awarded in research institutes. Precisely 49.7% strongly agree that funding of research will promote their career progression within research. Yet, 40.1% agree that they are considering leaving research to a non-research sector. However, 50.2% agree that they are considering leaving to another research sector. Notwithstanding, 45.0% strongly agree that there is a lack of appropriate infrastructure while 55.3% agree that there is a lack of steady electricity. Although, 43.5% disagree that there is a lack of collaborative research, about 66.2% agree that there is a lack of research interest by policymakers. Regrettably, 24.4% strongly agree that they place more value on meeting metrics than on quality research (Table 3).

## Discussion

Nigeria has about 517 tertiary and academic research institutions (Supplementary Material), yet it lags behind in economic development. Research in Nigeria’s tertiary and research institutions lack the priority it deserves due to inadequate funding. Scientists’ research productivity is regarded as a significant contribution to the production of knowledge and growth within any National Innovation System (NIS) (Salter and Martin, 2001). This study aimed to research the researcher and explore live experiences of the academic staff in Nigeria research and tertiary institutions without research funding. In light of the inadequate research productivity of academics, this study mainly depends on challenges and research productivity of academics in Nigeria research and tertiary institutions. The expected outcomes of functional research and development initiatives includes equipping researchers with the needed capacity with which to carry out their economic activities with a significant degree of expertise in order to achieve effective productivity. The major criterion for promotion is the number of peer-review articles authored by an academic staff member in many parts of the globe. Some academic staff may commit research misconduct due to the urgent need to publish in order to quickly reach the required publication threshold for promotion.

The study reveals that although there were more male than female academics in terms of numerical strength in this study, in actual sense, women were more productive in terms of the average number of publications (9.7 and 10.8 publications per male and female respondents, respectively). A previous finding confirms the gender gap in research productivity where female academics publish less on average than their male counterparts (Tower et al., 2007). This study found a gender influence, which is contrary to the report of Carr et al. (1998) which reveals a higher productivity among men but in agreement with the finding of Oyeyemi et al. (2019) who reported no gender influence.

Some of the challenges identified by this study have been established by other investigators elsewhere (Aslam et al., 2005; Dickson-swift et al., 2006; Baro and Ebhomeya, 2012; Okoduwa et al., 2018). Major challenges hindering research productivity of academics in Nigeria research and tertiary institutions as revealed by this study were non-funding of research, lack of mentorship, brain drain challenge, lack of training, lack of motivation, and non-payment of hazard and publication allowances. In all, a lack of funding of research is the hallmark of challenge affecting research tasks. A greater proportion of the participants reported non-funding of research. Many respondents indicated brain drain challenge; non-payment of hazard and publication allowances was also reported and lack of motivation. This implies that there is a lack of motivation and incentives for Nigeria researchers and many breakthrough research has been nearly swept under the carpet discouraging researchers and sending wrong signals to other aspiring researchers. The brain drain challenge revealed in this study is a major obstacle to the development of Africa resulting in the mass departure of intellectuals and skilled population to Western nations (Adefusika 2010). This observation was in accordance with the finding of Williams (2013). Similarly, Yusuf (2012) lamented the poor state of research funding and deteriorating research infrastructure as challenges facing research productivity of academic staff in Nigeria. Mentoring is also part of the problem facing academic staff in improving research productivity as rightly observed by Bentley (2012) and this has implications for managing tertiary and research institutes. This finding implies that a whole lot of predicaments confronting Nigeria’s tertiary/research institutions are orchestrated by challenges emanating from a lack of prioritization of research by policymakers to fund research in Nigeria.

On the perception of respondents with respect to research, the study revealed that a substantial number of respondents indicated that research is good, but policymakers do not prioritize research while some reported that the functionality of research determines development and others reported that research is demanding and impoverishes researchers due to non-funding. Obinna and Onuorah (2011) reported that a lack of interest and funding for research is responsible for the non-availability of accurate data in Nigeria. This study advocates for the need for policymakers in Nigeria to commit vigorously to an expanded role that better utilizes the expertise, knowledge, and resources at their disposal for the betterment of national development.

Results of this study indicate that demographic factors like academic qualifications and designation have a statistically significant correlation with research productivity. The study established that academic qualification is a very important variable that enhances the efficiency of academics with respect to research productivity. This supports the findings of Babalola (2014), who reported that academic qualification influences the productivity of researchers to a significant level, but contradicts that of Sarah et al. (2015) who reported that educational qualifications have no correlation with research productivity. Data obtained from this study also revealed that there is a correlation between age and research productivity. This finding does not conform with the results of Kotrlik et al. (2002) and Ramsden (1994) which reported that age has no correlation with research productivity.

The study reveals that there are significant differences in the number of conference papers with respect to academic disciplines. Academics in the sciences, medical sciences, and engineering had more conference papers than those in management sciences, education, and humanities. This could be because they have the benefit of getting more research funding and having more opportunities to present at scholarly conferences. This finding agrees with the viewpoint of previous studies which suggest that academics in natural sciences, engineering, and medical science publish more than their peers in humanities, social sciences, and business management (Jung, 2012; Kyvik 2003; Shin and Cummings, 2010). The low academic research output recorded by academics in the present study can be attributed to a lack of devoted time for carrying out research. This finding is consistent with those of previous Nigerian studies (Okiki 2013; Kpolovie et al, 2017) which reported a very low level of research productivity of academic staff. The low level of research productivity of researchers established in this study is in agreement with the findings of Yusuf (2012), University World News (2013), and Bassey et al. (2007). This low research output could perhaps reflect the low priority given to research and development by government and policymakers in Nigeria.

There is an indication that research productivity and academic degree are related. In this study, the research productivity of PhD holders was higher than those of other degree holders. The factor that could be responsible for this higher research productivity among PhD holders depends on the number of PhD participants, research collaboration, and the time devoted to research. This could be attributed to the fact that they have more opportunities to be productive in research and have greater confidence (Teodorescu, 2000). This assertion is similar to the findings of a study that showed that research collaboration increases productivity (Abramo et al., 2009). From the respondents’ point of view, they pinpointed factors that will motivate researchers and increase their productivity to include adequate funding of research, modern research facilities, equipment, and infrastructures, and payment of hazard and publication allowances, among others. A study reported that better remuneration and other monetary rewards could serve as a motivation for academics to participate actively in research (Nguyen, 2015). Somewhere, researchers argued that raising their self-esteem contributed to increased research productivity (van Lankveld et al., 2017).

Several studies establish a substantial relationship between higher research productivity and middle age (about 35–55 years) (Baldwin et al., 2005; Jung, 2014); while other studies revealed that more research productivity is associated with older age (Kwiek, 2018; Vuong et al., 2017). However, some other studies reported a decrease in research productivity with an increase in the age of academic staff (Smeby & Try, 2005; Albert et al., 2016). This study established higher research productivity among those in the age group of 31–40 with a decrease in productivity associated with older age. This finding did not give credence to the study of Ishola et al. (2018), which established that job experience has a significant impact on staff productivity. This finding demonstrated that the research productivity of respondents in sciences is overwhelmingly greater than those in management sciences, humanities, education, religious studies, and library studies. The study further revealed that 68.5% of the respondents accepted that adequate funding of research will promote the nation’s scientific and technological advancement. The study also reveals that a lack of research funding is a factor that limits researchers’ performance. Environmental factors such as institutional missions, leadership, rewards, orientation, availability of resources, and individual-institutional dichotomy could affect research productivity (Jung 2012; Quimbo and Salabu, 2014). Other factors that could have powerful effects on research performance of academics are government investment, national policies, politics, academic freedom, development partners, and support from industries and international donor agencies (Negash et al., 2019; Pornsalnuwat, 2014; Sam and Dahles, 2017; Tien, 2016).

In many African countries, the governments spend less than 1% of their total GDP on research and development, while about 90% of their funding for research is funded by bilateral and multilateral donors (Kraemer-Mbula and Scerri, 2015; Urama et al., 2015). It has been revealed that research collaboration with international colleagues is one of the most powerful links of high research productivity of scientists (Kwiek, 2016). More often than not, collaboration with international colleagues is a characteristic of productive scientists (Akbaritabar et al, 2018 *;* Kyvik and Reymert, 2017; Nguyen et al., 2017;, 2018; Kwiek, 2018; Vuong et al., 2019). Research funding has positive impacts on research productivity. It was observed in this study that the low level of productivity indicated by participants can be attributed to a lack of funding. This study suggests that funding of research can significantly increase researchers’ productivity. Hence, there is an urgent need for the Nigerian government to begin the prioritization of research and consider the needs of Nigerian researchers. Therefore, to achieve the Sustainable Development Goals by 2030, it is very significant to invest in research and research experts.

Non-funding of research has a substantial effect on researchers and it is the precursor of the challenges confronting Nigerian researchers as well as the development of the nation. There is an urgent need for the government of Nigeria to help researchers and the country in the aftermath of further developmental and societal catastrophes. There is a need for systemic changes in the way academic research is structured and supported so as to attract and retain the diversity of talent that is critical to address the current and future societal and developmental challenges in the country. Furthermore, the national government has a critical role to play in engaging all sectors in the research ecosystem to develop coordinated research workforce strategies, incentives to implement these strategies, and measures to monitor the activities in the sector. In the context of ‘building a better society’ from the COVID-19 pandemic, R&D can play a bigger role in moving the country and it requires more innovativeness. This will strengthen and tackle poverty which could have an insightful policy impact particularly on long-standing developmental challenges.

Also, this study strongly advocate policies directed at the promotion of R&D, innovation policy, technological prowess, and establishing support systems that can boost morale and enhance positive research through improved researcher capabilities and presumed sufficient national capacity. The government and policymakers should think about the increasing globalization of R&D spending and directly support scientific and technical research, through grant-providing agencies.

The challenge to government and policymakers is to boost R&D expenditure of the gross domestic product (GDP), encourage experimentation and a greater diversity of methods while simultaneously ensuring that an effective peer-review process is in place to guide research funding. This facet of R&D support has the potential to make a substantive difference to researchers’ performance. In the end, only when the neglect of R&D is discouraged by creating chaotic changes in who is responsible for funding of R&D sectors and by top-down administrative command for all decision making, will a determined and ideal country persist.

## Conclusions

This study investigated the productivity and challenges researchers faced in order to meet their job schedule. From this study, it is even more glaring that there is little or no funding of research in Nigeria due to a lack of interest by policymakers. Researchers use their salaries to conduct research, publish articles, and attend conferences before they are promoted resulting in external pressure expressed by the “publish or perish” axiom. Researchers are faced with brain drain challenge, lack of motivation, lack of training, and this could hinder the country’s achievement of sustainable development goals. A reasonable strategy the government can adopt could be to use a mix of policies to support R&D while taking pains to boost diverse and even competing approaches by research scientists receiving support in order to foster innovation.

### Research Limitations and Strengths

Despite these important findings, one limitation of this study is that analyses were based on self-reported and declared data as provided by the respondents. Secondly, the use of self-report evaluation tools could produce measurement bias and inaccurate estimates of reported numbers of research publications and conferences attended. Thirdly, respondents may present an untrue picture to the investigator. For instance, answering what they would like a situation to be rather than what the actual situation is. Finally, the authors of this study could not explore the majority of tertiary and research institutions in some of the geographical regions due to fund constraints, hence could not assess a significant proportion of tertiary and research institutions in Nigeria. This, therefore, limits institutional variability. However, the comprehensive list of these institutions is presented in the Supplementary Material. Also, this study provides significant insights into the productivity of academics as well as the impact of non-funding in research and tertiary institutions and can serve as a model for future larger studies in Nigeria.

## Data Availability

The raw data supporting the conclusions of this article will be made available by the authors, without undue reservation.
