# Supplementary material for: Focused Research on the Challenges and Productivity of Researchers in Nigerian Academic Institutions Without Funding
Source: Front Res Metr Anal. 2021 Oct 28;6:727228. doi: 10.3389/frma.2021.727228 (PMC8596491; doi:10.3389/frma.2021.727228)
Supplement: Supplementary file 1 [file DataSheet1.pdf]

This is the SUPPLEMENTARY MATERIAL for Focused Research on the Challenges and Productivity of Researchers in Nigerian Academic Institutions without Funding

Citation: Igiri BE, Okoduwa SIR, Akabuogu EP, Okoduwa UJ, Enang IA, Idowu OO, Abdullahi S, Onukak IE, Onuruka CC, Christopher OPO, Salawu AO, Chris AO, and Onyemachi DI (2021) Research Study on the Challenges and Productivity of Researchers in Nigeria Academic Institutions Without Funding. Front. Res. Metr. Anal. 6:727228. doi: 10.3389/frma.2021.727228

Correspondence:
Stanley I.R. Okoduwa
siroplc@gmail.com

Highlight of the Tertiary and Academic Research Institutions in Nigeria

The strategic players in research and development (R&D) in Nigeria are the research-focused institutions, tertiary education system, private research establishments, and government agencies with R&D mandates. Presently, there are 197 universities (NUC, 2021a,b,c) with varying capacities for pure and applied R&D, 140 polytechnics with modest capacities in applied research (NBTE, 2021a,b,c), 89 colleges and specialized technological institutions (NCCE, 2021), 25 monotechnics (NBTE, 2021a,b,c), and 66 research institutes (FMARD, 2021; Nigerian Guide, 2021) with R & D programs targeted at the different sectors of the economy with a few recording some measure of success in product and process innovation. A simple random sampling of these institutions was conducted in a focused research in order to examine the challenges and productivity of researchers in Nigerian academic institutions without funding. The findings obtained in the research is documented in the main article which is available at doi: 10.3389/frma.2021.727228

	CONTENT	PAGE
1.	Statistics of tertiary academic institutions in Nigeria	2
2.	Research institutions in Nigeria	3
3.	Federal universities in Nigeria	4
4.	State universities in Nigeria	5
5.	Private universities in Nigeria	6
6.	Federal polytechnics in Nigeria	10
7.	State polytechnics in Nigeria	10
8.	Private polytechnics in Nigeria	11
9.	Federal colleges in Nigeria	12
10.	State colleges in Nigeria	13
11.	Private colleges in Nigeria	14
12.	Federal monotechnics in Nigeria	15
13.	State monotechnics in Nigeria	15
14.	Private monotechnics in Nigeria	15

STATISTICS OF TERTIARY ACADEMIC INSTITUTIONS IN NIGERIA

Table S1 | Summary of the Tertiary Academic Institutions in Nigeria

	Universities	Polytechnics	Monotechnics	Colleges of Education
Federal	45	31	21	22
State	53	48	2	47
Private	99	61	2	20
TOTAL	**197**	**140**	**25**	**89**

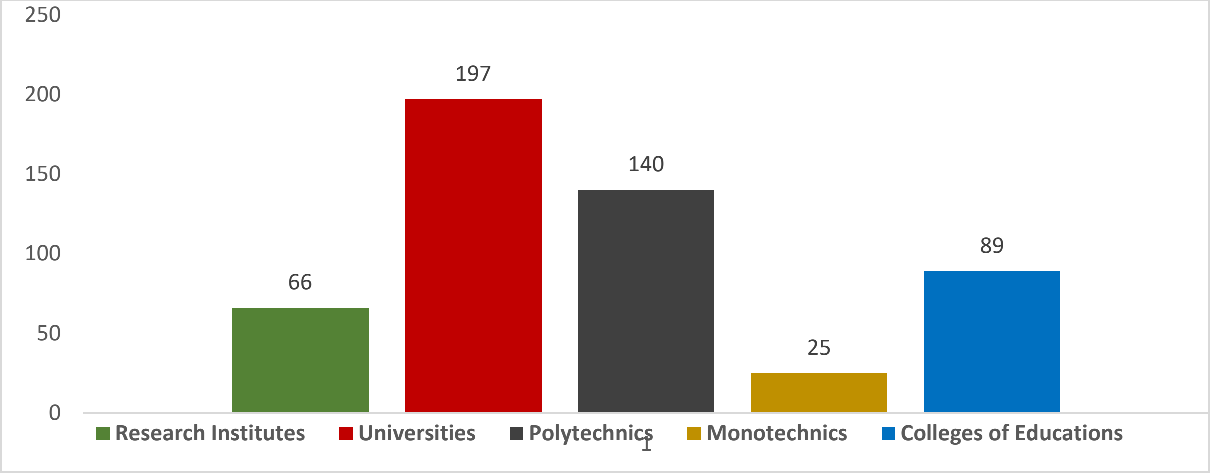

FIGURE S1 | Distribution of tertiary academic institutions in Nigeria

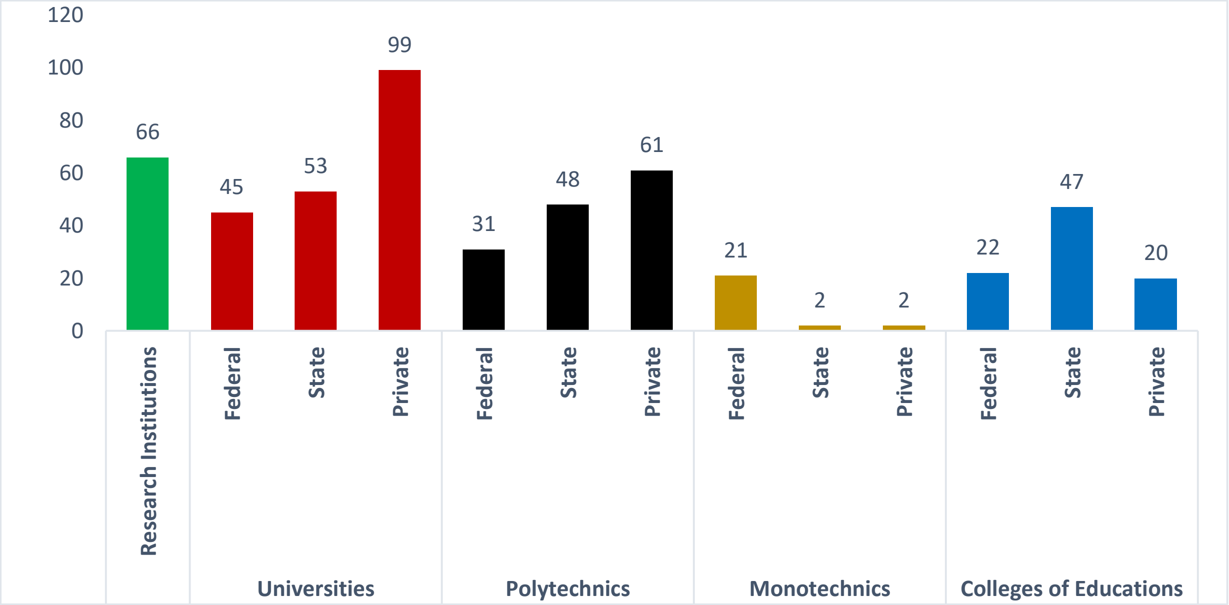

Figure S2 | Statistics of the Tertiary Academic Institutions in Nigeria

Table S2 | List of Research Institutions in Nigeria (CWN, 2021; FMARD, 2021; Nigerian Guide, 2021)

S/N	Names of Research Institutions in Nigeria
**1**	Agricultural Research Council of Nigeria (ARCN)
**2**	Agricultural Rural Management Training Institute (ARMTI), Ilorin
**3**	Animal Health Research Institutes (AHRI)
**4**	Animal Production, Fisheries and Oceanography Research Institutes (APFORI)
**5**	Arable Crops Research Institutes (ACRI)
**6**	Center for Energy Research and Development (CERD)
**7**	Centre for Energy Research and Training (CERT)
**8**	Centre for Management Development (CMD)
**9**	Cocoa Research Institute of Nigeria, Ibadan, (CRIN), Oyo
**10**	Energy Commission of Nigeria (ECN)
**11**	Federal College of Freshwater Fisheries Technology, (FCFFT) New Bussa
**12**	Federal Institute of Industrial Research (FIIRO), Oshodi, Lagos
**13**	Federal Ministry of Agriculture (FMA)
**14**	Federal Ministry of Science, Technology and Innovation (FMSTI)
**15**	Federal Ministry of Water Resources (FMWR)
**16**	Forestry Research Institute of Nigeria (FRIN), Ibadan
**17**	Forestry, Horticulture and Tree Crops Research Institutes (FHTCRI)
**18**	Institute for Advanced Medical Research and Training (IAMRT)
**19**	Institute for Agricultural Research (IAR)
**20**	Institute of Agricultural Research & Extension Services, (IAR & ES) Ahmadu Bello University, Zaria
**21**	Institute of Agricultural Research & Training, (IAR & T) Obafemi Awolowo University, Ibadan
**22**	Institute of Archaeology and Museum Studies (IAMS)
**23**	Institute of Chartered Chemists of Nigeria (ICCON)
**24**	Institute of Child Health (ICH)
**25**	Institute of Human Virology (IHV), Abuja
**26**	Institute of Operations Research of Nigeria (INFORN)
**27**	International Institute of Tropical Agriculture (IITA)
**28**	International Livestock Research Institute (ILRI)
**29**	Lake Chad Research Institute (LCRI)
**30**	National Agency for Food and Drug Administration and Control (NAFDAC)
**31**	National Agency for Science & Engineering Infrastructure (NASENI), Abuja
**32**	National Agricultural Extension Research and Liaison Services (AERLS)
**33**	National Animal Production Research Institute, (NAPRI) Zaria
**34**	National Center for Agricultural Mechanization (NCAM)
**35**	National Center for Energy Research and Development (NCERD)
**36**	National Center for Technology Management (NCTM)
**37**	National Centre for Genetic Resources and Biotechnology (NACGRAB)
**38**	National Cereals Research Institute (NCRI)
**39**	National Horticultural Research Institute (NIHORT)
**40**	National Institute for Pharmaceutical Research and Development (NIPRD)
**41**	National Institute of Freshwater Fisheries Research, New Bussa
**42**	Nigerian Institute of Leather and Science Technology (NILEST)
**43**	National Research Institute for Chemical Technology (NARICT)

**44**	National Root Crops Research Institute (NRCI)
**45**	National Space Research and Development Agency (NASRDA)
**46**	National Veterinary Research Institute (NVRI)
**47**	Nigeria Institute of Science Laboratory (NISLT)
**48**	Nigerian Academy of Science (NAS)
**49**	Nigerian Building and Road Research Institute (NBRRI)
**50**	Nigerian Educational Research Council (NERC)
**51**	Nigerian Institute for Oceanography and Marine Research (NIOMR)
**52**	Nigerian Institute for Oil Palm Research (NIFOR), Benin City Edo State
**53**	Nigerian Institute for Trypanosomiasis Research (NITR)
**54**	Nigerian Institute of Food Science and Technology (NIFST)
**55**	Nigerian Institute of Medical Research (NIMR)
**56**	Nigerian Institute of Social and Economic Research (NISER)
**57**	Nigerian Stored Products Research Institute (NSPRI)
**58**	Projects Development Institute (PRODA)
**59**	Raw Materials Research and Development Council (RMRDC)
**60**	Research Institutes Organisations in Nigeria (RION)
**61**	Rubber Research Institute of Nigeria (RRIN), Iyanomo, Benin City Edo State
**62**	Scientific Equipment Development Institute (SEDI-E)
**63**	Sheda Science and Technology Complex (SHESTCO)
**64**	Social Sciences Academy of Nigeria (SSAN)
**65**	Sokoto Energy Research Centre (SERC)
**66**	Unilorin Sugar Research Institute (USRI)

Table S3 | List of Federal Universities in Nigeria (NUC, 2021a)

S/N	Names of Federal Universities in Nigeria	Year Established
**1**	Abubakar Tafawa Balewa University, (ATBU) Bauchi	1988
**2**	Ahmadu Bello University, (ABU) Zaria	1962
**3**	Bayero University, Kano	1975
**4**	Federal University Gashua, Yobe	2013
**5**	Federal University of Petroleum Resources, Effurun	2007
**6**	Federal University of Technology, Akure	1981
**7**	Federal University of Technology, Minna	1982
**8**	Federal University of Technology, Owerri	1980
**9**	Federal University, Dutse, Jigawa State	2011
**10**	Federal University, Dutsin-Ma, Katsina	2011
**11**	Federal University, Kashere, Gombe State	2011
**12**	Federal University, Lafia, Nasarawa State	2011
**13**	Federal University, Lokoja, Kogi State	2011
**14**	Federal University, Ndifu-Alike, Ebonyi State	2011
**15**	Federal University, Otuoke, Bayelsa	2011
**16**	Federal University, Oye-Ekiti, Ekiti State	2011
**17**	Federal University, Wukari, Taraba State	2011
**18**	Federal University, Birnin Kebbi	2013
**19**	Federal University, Gusau Zamfara	2013
**20**	Michael Okpara University of Agricultural Umudike	1992

**21**	Modibbo Adama University of Technology, Yola	1981
**22**	National Open University of Nigeria, Lagos	2002
**23**	Nigeria Police Academy Wudil	2013
**24**	Nigerian Defence Academy Kaduna	1985
**25**	Nnamdi Azikiwe University, Awka	1992
**26**	Obafemi Awolowo University,Ile-Ife	1962
**27**	University of Abuja, Gwagwalada	1988
**28**	Federal University of Agriculture, Abeokuta	1988
**29**	University of Agriculture, Makurdi	1988
**30**	University of Benin	1970
**31**	University of Calabar	1975
**32**	University of Ibadan	1948
**33**	University of Ilorin	1975
**34**	University of Jos	1975
**35**	University of Lagos	1962
**36**	University of Maiduguri	1975
**37**	University of Nigeria, Nsukka	1960
**38**	University of Port-Harcourt	1975
**39**	University of Uyo	1991
**40**	Usumanu Danfodiyo University	1975
**41**	Nigerian Maritime University Okerenkoko, Delta State	2018
**42**	Air Force Institute of Technology, Kaduna	2018
**43**	Nigerian Army University Biu	2018
**44**	University of Health Technology, Otukpo Benue State	2020
**45**	Federal University of Agriculture, Zuru, Kebbi State	2020

Table S4 | List of State Universities in Nigeria (NUC, 2021c)

S/N	Names of State Universities in Nigeria	Year Established
**1**	Abia State University, Uturu	1981
**2**	Adamawa State University Mubi	2002
**3**	Adekunle Ajasin University, Akungba	1999
**4**	Akwa Ibom State University of Technology, Uyo	2010
**5**	Ambrose Alli University, Ekpoma	1980
**6**	Chukwuemeka Odumegwu Ojukwu University, Uli	2000
**7**	Bauchi State University, Gadau	2011
**8**	Benue State University, Makurdi	1992
**9**	Yobe State University, Damaturu	2006
**10**	Cross River State University of Science &Technology, Calabar	2004
**11**	Delta State University Abraka	1992
**12**	Ebonyi State University, Abakaliki	2000
**13**	Ekiti State University	1982
**14**	Enugu State University of Science and Technology, Enugu	1982
**15**	Gombe State Univeristy, Gombe	2004
**16**	Ibrahim Badamasi Babangida University, Lapai	2005
**17**	Ignatius Ajuru University of Education, Rumuolumeni	2010
**18**	Imo State University, Owerri	1992

**19**	Sule Lamido University, Kafin Hausa, Jigawa	2013
**20**	Kaduna State University, Kaduna	2004
**21**	Kano University of Science & Technology, Wudil	2000
**22**	Kebbi State University, Kebbi	2006
**23**	Kogi State University Anyigba	1999
**24**	Kwara State University, Ilorin	2009
**25**	Ladoke Akintola University of Technology, Ogbomoso	1990
**26**	Ondo State University of Science and Technology Okitipupa	2008
**27**	River State University of Science and Technology	1979
**28**	Olabisi Onabanjo University, Ago Iwoye	1982
**29**	Lagos State University, Ojo	1983
**30**	Niger Delta University Yenagoa	2000
**31**	Nasarawa State University Keffi	2002
**32**	Plateau State University Bokkos	2005
**33**	Tai Solarin University of Education Ijebu Ode	2005
**34**	Umar Musa Yar' Adua University Katsina	2006
**35**	Osun State University Osogbo	2006
**36**	Taraba State University, Jalingo	2008
**37**	Sokoto State University	2009
**38**	Northwest University Kano	2012
**39**	Oyo State Technical University Ibadan	2012
**40**	Ondo State University of Medical Sciences	2015
**41**	Edo University Iyamo	2016
**42**	Eastern Palm University Ogboko, Imo State	2016
**43**	University of Africa Toru Orua, Bayelsa State	2016
**44**	Bornu State University, Maiduguri	2016
**45**	Moshood Abiola University of Science and Technology Abeokuta	2017
**46**	Gombe State University of Science and Technology	2017
**47**	Zamfara State University	2018
**48**	Bayelsa Medical University	2019
**49**	Confluence University of Science and Technology, Osara,	2020
**50**	University of Delta, Agbor	2021
**51**	Delta University of Science and Technology, Ozoro	2021
**52**	Dennis Osadebe University, Asaba	2021
**53**	King David University of Medical Sciences, Uburu, Ebonyi State	2021

Table S5 | List of Private Universities in Nigeria (NUC, 2021b)

S/N	Names of Private Universities in Nigeria	Year Established
**1**	Achievers University, Owo	2007
**2**	Adeleke University, Ede	2011
**3**	Afe Babalola University, Ado-Ekiti – Ekiti State	2009
**4**	African University of Science & Technology, Abuja	2007
**5**	Ajayi Crowther University, Ibadan	2005
**6**	Al-Hikmah University, Ilorin	2005

**7**	Al-Qalam University, Katsina	2005
**8**	American University of Nigeria, Yola	2003
**9**	Augustine University	2015
**10**	Babcock University,Ilishan-Remo	1999
**11**	Baze University	2011
**12**	Bells University of Technology, Otta	2005
**13**	Benson Idahosa University, Benin City	2002
**14**	Bingham University, New Karu	2005
**15**	Bowen University, Iwo	2001
**16**	Caleb University, Lagos	2007
**17**	Caritas University, Enugu	2005
**19**	Chrisland University	2015
**20**	Covenant University Ota	2002
**21**	Crawford University Igbesa	2005
**22**	Crescent University	2005
**23**	Edwin Clark University, Kaigbodo	2015
**24**	Elizade University, Ilara-Mokin	2012
**25**	Evangel University, Akaeze	2012
**26**	Fountain Unveristy, Oshogbo	2007
**27**	Godfrey Okoye University, Ugwuomu-Nike – Enugu State	2009
**28**	Gregory University, Uturu	2012
**29**	Hallmark University	2015
**30**	Hezekiah University, Umudi	2015
**31**	Igbinedion University Okada	1999
**32**	Joseph Ayo Babalola University, Ikeji-Arakeji	2006
**33**	Kings University	2015
**34**	Kwararafa University, Wukari	2005
**35**	Landmark University, Omu-Aran.	2011
**36**	Lead City University, Ibadan	2005
**37**	Madonna University, Okija	1999
**38**	Mcpherson University, Seriki Sotayo, Ajebo	2012
**39**	Micheal & Cecilia University	2015
**40**	Mountain Top University	2015
**41**	Nile University of Nigeria, Abuja	2009
**42**	Novena University, Ogume	2005
**43**	Obong University, Obong Ntak	2007
**44**	Oduduwa University, Ipetumodu – Osun State	2009
**45**	Pan-Atlantic University, Lagos	2002
**46**	Paul University, Awka – Anambra State	2009
**47**	Redeemer’s University, Mowe	2005
**48**	Renaissance University, Enugu	2005
**49**	Rhema University, Obeama-Asa – Rivers State	2009
**50**	Ritman University	2015
**51**	Salem University, Lokoja	2007
**52**	Samuel Adegboyega University, Ogwa.	2011
**53**	Southwestern University, Oku Owa	2012
**54**	Summit University	2015
**55**	Tansian University, Umunya	2007

**56**	University of Mkar, Mkar	2005
**57**	Veritas University	2007
**58**	Wellspring University, Evbuobanosa – Edo State	2009
**59**	Wesley University. of Science & Technology, Ondo	2007
**60**	Western Delta University, Oghara Delta State	2007
**61**	Christopher University Mowe	2015
**62**	Kola Daisi University Ibadan, Oyo State	2016
**63**	Anchor University Ayobo Lagos State	2016
**64**	Dominican University Ibadan Oyo State	2016
**65**	Legacy University, Okija Anambra State	2016
**66**	Arthur Javis University Akpoyubo Cross river State	2016
**67**	Cn Hill University Eiyenkorin, Kwara State	2016
**68**	Coal City University Enugu State	2016
**68**	Clifford University Owerrinta Abia State	2016
**69**	Admiralty University, Ibusa Delta State	2017
**70**	Spiritan University, Nneochi Abia State	2017
**71**	Precious Cornerstone University, Oyo	2017
**72**	PAMO University of Medical Sciences, Portharcourt	2017
**73**	Atiba University Oyo	2017
**74**	Eko University of Medical and Health Sciences Ijanikin, Lagos	2017
**75**	Skyline University, Kano	2018
**76**	Greenfield University, Kaduna	2019
**77**	Dominion University Ibadan, Oyo State	2019
**78**	Trinity University Ogun State	2019
**79**	Westland University Iwo, Osun State	2019
**80**	Topfaith University, Mkpatak, Akwa Ibom State	2021
**81**	Thomas Adewumi University, Oko-Irese, Kwara State	2021
**82**	Maranatha University, Mgbidi, Imo State	2021
**83**	Ave Maria University, Piyanko, Nasarawa State	2021
**84**	Al-Istiqama University, Sumaila, Kano State	2021
**85**	Mudiame University, Irrua, Edo State	2021
**86**	Havilla University, Nde-Ikom, Cross River State	2021
**87**	Claretian University of Nigeria, Nekede, Imo State	2021
**88**	NOK University, Kachia, Kaduna State	2021
**89**	Karl-Kumm University, Vom, Plateau State	2021
**90**	James Hope University, Lagos, Lagos State	2021
**91**	Maryam Abacha American University of Nigeria, Kano State	2021
**92**	Capital City University, Kano State	2021
**93**	Ahman Pategi University, Kwara State	2021
**94**	University of Offa, Kwara State	2021
**95**	Mewar University, Masaka, Nasarawa State	2021
**96**	Edusoko University, Bida, Niger State	2021
**97**	Philomath University, Kuje, Abuja	2021
**98**	Khadija University, Majia, Jigawa State	2021
**99**	Anan University, Kwall, Plateau State	2021

Table S6 | List of Federal Polytechnics in Nigeria (NBTE, 2021a)

S/N	Names of Federal Polytechnics in Nigeria	Year Established
**1**	Akanu Ibiam Federal Polytechnic Unwana, Afikpo, Ebonyi State.	1981
**2**	Auchi Polytechnic, Auchi, Edo State.	1973
**3**	Federal Polytechnic Ado Ekiti, Ekiti State.	1977
**4**	Federal Polytechnic Bali, Taraba State.	2007
**5**	Federal Polytechnic Bauchi, Bauchi State.	1979
**6**	Federal Polytechnic Bida, Niger State.	1977
**7**	Federal Polytechnic Damaturu, Yobe State.	1993
**8**	Federal Polytechnic, Daura, Katsina State	2019
**9**	Federal Polytechnic Ede, Osun State.	1992
**10**	Federal Polytechnic Ekowe, Bayelsa State	2007
**11**	Federal Polytechnic Idah, Kogi State.	1977
**12**	Federal Polytechnic Ilaro, Ogun State.	1979
**13**	Federal Polytechnic Ile-Oluji, Ondo State	2016
**14**	Federal Polytechnic Kaura Namoda, Zamfara State.	1983
**15**	Federal Polytechnic Kaltungo, Gombe State	2019
**16**	Federal Polytechnic Mubi, Adamawa State.	1979
**17**	Federal Polytechnic Nasarawa, Nasarawa State.	1983
**18**	Federal Polytechnic Nekede, Owerri, Imo State.	1977
**19**	Federal Polytechnic Offa, Kwara State.	1992
**20**	Federal Polytechnic Oko, Anambra State.	1982
**21**	Federal Polytechnic of Oil and Gas Bonny, Rivers State	2014
**22**	Federal Polytechnic Ukana, Akwa Ibom State	2014
**23**	Hussaini Adamu Federal Polytechnic, Kazaure Jigawa State.	1991
**24**	Kaduna Polytechnic, Kaduna.	1956
**25**	National Institute of Construction Technology Uromi	2014
**26**	Waziri Umaru Federal Polytechnic, Birnin Kebbi.	1976
**27**	Yaba College of Technology, Yaba, Lagos State.	1947
**28**	Airforce Institute of Technology (AFIT), NAF Base Kaduna	1977
**29**	Petroleum Training Institute Effurun, Effurun. Delta State	1972
**30**	Nigerian Army Institute of Technology and Environmental Science (NAITES) Makurdi	1960
**31**	Nigerian College of Aviation Technology (NCAT), Sokoto Road, Zaria,	

Table S7 | List of State Polytechnics in Nigeria (NBTE, 2021c)

S/N	Names of State Polytechnics in Nigeria	Year Established
**1**	Abdu Gusau Polytechnic, Talata Mafara, Talata Mafara, Zamfara State.	1992
**2**	Abia State Polytechnic, Aba, Abia State.	1992
**3**	Abraham Adesanya Polytechnic, Dogbolu/Akanran Ibadan Road, Atikori, Ijebu-Igbo, Ogun State.	2004

**4**	Abubakar Tatari Ali Polytechnic, Bauchi, Jos Rd, Bauchi, Bauchi State.	1988
**5**	Adamawa State Polytechnic, Yola, Adamawa State.	1991
**6**	Akwa Ibom State College of Art & Science, Nung Ukim, Akwa Ibom State.	1997
**7**	Akwa Ibom State Polytechnic,Ikot Osurua, Ikot -Ekpene,Akwa Ibom State.	1991
**8**	Bayelsa State College of Arts and Science, Elebele, Yenogoa	2002
**9**	Benue State Polytechnic, Ugbokolo, Ugbokolo, Benue State.	1976
**10**	Binyaminu Usman Polytechnic, Hadejia,Jigawa State	2016
**11**	D.S. Adegbenro ICT Polytechnic, Itori-Ewekoro, Ifo, Ogun State.	2004
**12**	Delta State Polytechnic, Ogwashi-Uku, Ogwashi-Uku,Delta State	2002
**13**	Delta State Polytechnic, Otefe-Oghara, Otefe-Oghara, Delta State	2002
**14**	Delta State Polytechnic, Ozoro, Ozoro, Delta State.	2003
**15**	Delta State School of Marine Technology, Burutu, Warri, Delta State.	2017
**16**	Edo State Institute of Technology and Management, Usen, Benin City, Edo State.	2002
**17**	Enugu State Polytechnic, Iwollo	2017
**18**	Gateway Polytechnic, Isara Ode-Remo, Ogun State.	2004
**19**	Hassan Usman Katsina Polytechnic (HUK), Katsina	1983
**20**	Ibarapa Polytechnic, Eruwa, Oyo State	2013
**21**	Imo State Polytechnic, Umuagwo, Ohaji, , Umuagwo, Ohaji Owerri, Imo State	1978
**22**	Institute of Management and Technology, Enugu, Enugu State.	1965
**23**	Institute of Technology and Management (ITM), Ugep, Cross River State.	2012
**24**	Jigawa State Polytechnic, Dutse, Jigawa State.	1991/2008
**25**	Kano State Polytechnic, Kano, Kano State.	1976
**26**	Ken Sarowiwa Polytechnic, Bori, Rivers State.	1988
**27**	Kogi State Polytechnic, Lokoja, Kogi State.	1993
**28**	Kwara State Polytechnic, Ilorin, Kwara State.	1973
**29**	Lagos State Polytechnic, Ikorodu, Ikeja, Lagos, Lagos State.	1977
**30**	Mai-Idris Alooma Polytechnic, Geidam, Yobe State.	2002
**31**	Moshood Abiola Polytechnic, Abeokuta (Converted to State University in 2017), Abeokuta, Ogun State.	1979
**32**	Nasarawa State Polytechnic, Lafia, Nasarawa State.	2001
**33**	Niger State Polytechnic, Zungeru, Niger State.	1991
**34**	Nuhu Bamalli Polytechnic, Zaria, Kaduna State.	1989
**35**	Ogun State Institute of Technology, Igbesa, Ogun State.	2004
**36**	Ogun State Polytechnic, Ipokia	2017
**37**	Oke-Ogun Polytechnic, Shaki, Oyo State	2013
**38**	Osun State College of Technology, Esa – Oke, Osun State.	1991
**39**	Osun State Polytechnic, Iree, Osun State.	1992
**40**	Oyo State College of Agriculture and Technology, Igbo Ora	2018
**41**	Plateau State Polytechnic, Barkin-Ladi, Jos, Plateau State.	1978

**42**	Port-Harcourt Polytechnic, Port-Harcourt,Rivers State.	1991
**43**	Ramat Polytechnic, Maiduguri, Borno State.	1976
**44**	Rufus Giwa Polytechnic, Owo, Ondo State.	1979
**45**	Taraba State Polytechnic, Suntai	2017
**46**	The Polytechnic Ibadan, Ibadan, Oyo State.	1970
**47**	Umaru Ali Shinkafi Polytechnic, Sokoto, Sokoto State.	2000
**48**	Zamfara State College of Arts and Science, Gusau	2000

Table S8 | List of Private Polytechnics in Nigeria (NBTE, 2021b)

S/N	Names of Private Polytechnics in Nigeria	Year Established
**1**	Al-Hikma Polytechnic Karu, Mararaba Gurku, Karu,Nasarawa	2016
**2**	Allover Central Polytechnic, Sango Ota Ogun State	1998/2003
**3**	Ajayi Polytechnic Ikere Ekiti, Ekiti State	2017
**4**	Ashi PolytechnicAnyiin, Benue State	2018
**5**	Best Solution Polytechnic, Akure Akure – Ondo State	2016
**6**	Bolmor Polytechnic, Dugbe, Ibadan, Oyo State	2015
**7**	Calvary Polytechnic, Owa-OyibuDelta State	2016
**8**	Citi Polytechnic Dutse-Dawaki Road Dutse Alhaji FCT	2018
**9**	Coastal Polytechnic,Apapa Lagos	2020
**10**	Covenant Polytechnic Abayi, AbaAbia State.	2010
**11**	Crown Polytechnic, Odo, Ekiti State	2008
**12**	Dorben Polytechnic, Abuja	1995/2008
**13**	Eastern Polytechnic Rivers State	2015
**14**	Enville Institute of Management and Technology, Itokin, Lagos	2019
**15**	Fidei Polytechnic, Gboko-Aliade Road, Benue State.	2007
**16**	Gboko Polytechnic, Gboko, Benue State	2016
**17**	Global Polytechnic, Akure, Ondo State	2016
**18**	Grace Polytechnic, Surulere, Lagos	1962/1999
**19**	Graceland Polytechnic, Offa, Kwara State	2018
**20**	Grundtvig Polytechnic Oba Anambra State	2019
**21**	Harry Pass Polytechnic Tse Usen Mkar Gboko Benue State	2020
**22**	Heritage Polytechnic, Ikot Udota, Eket,Akwa Ibom State	2010
**23**	Ibadan City Polytechnic, Ibadan Oyo	2015
**24**	Igbajo Polytechnic, Igbajo, Osun State.	2009
**25**	Interlink Polytechnic, Ijebu-Jesa, Osun State	2008
**26**	Kalac Christal Polytechnic, Lekki, Lagos State	2014
**27**	Kings Polytechnic, Ubiaja, Edo State	2010
**28**	Landmark Polytechnic, Ayetoro/Itele Ogun State	2018
**29**	Lagos City Polytechnic, Ikeja,	1990/1995
**30**	Lens Polytechnic, Offa, Kwara State	2016
**31**	Lighthouse Polytechnic, Benin City, Edo State	2008
**32**	Marist Polytechnic, Umuchigbo, Iji-Nike, Emene Enugu State	2016
**33**	Mater Dei PolytechnicUgwuoba TownOji River LGAEnugu State	2018
**34**	Nacabs Polytechnic, AkwangaP.M.B. 001 Akwanga, Nasarawa State	2013
**35**	Nogak Polytechnic, Ikom,Cross Rivers State	2012

**36**	Novelty Polytechnic, Kishi Kishi Oyo	2019
**37**	Our Saviour Institute of Science, Agriculture & Technology, Enugu State.	1989/1991
**38**	Prime Polytechnic, Jida Bassa, Ajaokuta, Kogi	2015
**39**	Redeemers College of Technology & Management, Mowe, Ogun State	2018
**40**	Ronik Polytechnic, Ejigbo, Ikeja, Lagos, Lagos	2001/2003
**41**	Saf Polytechnic, Iseyin Oyo State.	2018
**42**	Savanah Institute of Technology, Abakaliki, Ebonyi	2017
**43**	Shaka Polytechnic, Benin City, Edo State.	2013
**44**	Speedway Polytechnic,Osoba Ojudu, Ogun State	2019
**45**	St. Mary Polytechnic, Kwamba-Suleja, Niger State	2015
**46**	Stars Polytechnic Ota Ogun	2020
**47**	Southern Atlantic Polytechnic, Uyo – Akwa Ibom State	2020
**48**	Sure Foundation Polytechnic, Ikot Akai, Ibom State	2016
**49**	Temple Gate Polytechnic, Abayi, Osisioma, Abia State.	2009
**50**	The Polytechnic, Igbo-Owu, Kwara State	2015
**51**	The Polytechnic, Ile-Ife, Osun State.	1994/1999
**52**	The Polytechnic Iresi (Formerly College of Technology, Iresi) Osun State	2014
**53**	The Polytechnic Otada Adoka, Otukpo, Benue State	2016
**54**	Timeon Kairos Polytechnic Lagos	2019
**55**	Tower Polytechnic, Ibadan	2010
**56**	Trinity Polytechnic Uyo,Akwa Ibom State	2016
**57**	Uma Ukpai Polytechnic,Asaga Ohafia, Abia State	2017
**58**	Uyo City Polytechnic, Akwa Ibom State	2014
**59**	Valley View Polytechnic, Ohafia Abia State	2016
**60**	Villanova Polytechnic, Imesi-Ile (Formerly The Polytechnic, Imesi-Ile) Osun State.	2013
**61**	Wolex Polytechnic, Osun State	1996/1

Table S9 | List of Federal Colleges of Education in Nigeria (NBTE, 2021a; NCCE, 2021)

S/N	Names of Federal Colleges of Education in Nigeria	Location
**1**	Federal College of Education (Technical), Asaba	Asaba, Delta State.
**2**	Federal College of Education, Kano	Kano City, Kano State.
**3**	Federal College of Education (Special), Oyo	Oyo, Oyo State.
**4**	Federal College of Education, Abeokuta	Abeokuta, Ogun State
**5**	Federal College of Education, Eha-Amufu	Eha Amufu, Enugu State.
**6**	Federal College of Education (Technical), Gombe	Gombe, Gombe State.
**7**	Federal College of Education, Kontagora	Kontagora, Niger State.
**8**	Federal College of Education, Okene	Okene, Kogi State.
**9**	Federal College of Education (Technical), Omoku	OMoku, Rivers State.
**10**	Federal College of Education (Tech), Potiskum	Potiskum, Yobe State
**11**	Alvan Ikoku College of Education, Owerri	Owerri, Imo State.
**12**	Federal College of Education (Technical), Akoka	Akoka, Lagos State.
**13**	Federal College of Education (Technical), Bichi	Bichi, Kano State
**14**	Federal College of Education (Technical), Gusau	Gusau, Zamfara State.

**15**	Federal College of Education, Katsina	Katsina, Katsina State.
**16**	Federal College of Education, Obudu	Obudu, Cross River State.
**17**	Adeyemi College of Education, Ondo	Ondo, Ondo State.
**18**	Federal College of Education, Pankshin	Pankshin, Plateau State.
**19**	Federal College of Education, Yola	Yola, Adamawa State.
**20**	Federal College of Education, Zaria10.	Zaria, Kaduna State.
**21**	Nigerian Army School of Education (NASE), Ilorin	Ilorin, Kwara State.
**22**	Federal College of Education (T), Umunze	Umunze

Table S10 | List of State Colleges of Education in Nigeria (NBTE, 2021b; NCCE, 2021)

S/N	Names of State Colleges of Education in Nigeria	Location
**1**	College of Education, Gindiri	Gindiri, Plateau State.
**2**	Adamawa State College of Education, Hong	Yola, Adamawa State.
**3**	Tai Solarin College of Education, Ijebu-Ode	Ijebu-Ode, Ogun State.
**4**	College of Education, Ikere-Ekiti	Ikere-Ekiti, Ekiti State.
**5**	Ebonyi State College of Education, Ikwo	Ikwo, Ebonyi State.
**6**	College of Education, Warri	Edjeba Road, Warri, Delta State
**7**	FCT College of Education, Zuba	Zuba, Garki, FCT Abuja.
**8**	Osisatech College of Education, Enugu	Enugu, Enugu State.
**9**	Nasarrawa State College of Education, Akwanga	Akwanga, Nassarawa State.
**10**	Isa Kaita College of Education, Dutsin-Ma	Dutsin-Ma, Katsina State.
**11**	College of Education, Ekiadolor-Benin	Ekiadolor-Benin, Edo State
**12**	College of Education, Gashua, Damaturu	Gashua, Yobe state.
**13**	Kaduna State College of Education, Gidan-Waya, Kafanchan	Kafanchan, Kaduna State.
**14**	Osun State College of Education, Ilesa	Ilesa, Osun State.
**15**	Kwara State College of Education, Ilorin	Ilorin, Kwara State.
**16**	Kwara State College of Education	Oro, Kwara State.
**17**	College of Education, katsina-Ala	Katsina-Ala, Benue State.
**18**	Sa’adatu Rimi College of Education, Kumbotso, Kano	Kumbotso, Kano State.
**19**	College of Education (Technical), Lafiagi	Lafiagi, Kwara State.
**20**	Nwafor Orizu College of Education, Nsugbe	Nsugbe, Anambra State.
**21**	Adeniran Ogunsanya College of Education, Otto/Ijanikin	Otto/Ijanikin, Lagos State.
**22**	Emmanuel Alayande College of Education (EACOED), Oyo	Oyo, Oyo State.
**23**	College of Education, Waka BIU	Waka BIU, Borno State.
**24**	St. Augustine College of Education (Project Time), Lagos	Yaba, Lagos State.
**25**	Delta State College of Education, Agbor	Agbor, Delta State
**26**	Akwa Ibom State College of Education, Afahansit	Afahansit, Akwa Ibom State.
**27**	Kogi State College of Education, Ankpa	Ankpa, Kogi state.
**28**	Adamu Augie College of Education, Argungu	Argungu, Kebbi State.
**29**	College of Education, Azare	Azare, Bauchi State

**30**	Umar Ibn Ibrahim El-Kanemi College of Education, Science and Technology, Bama	Bama, Borno State.
**31**	College of Education, Jalingo	Jalingo, Taraba State.
**32**	Zamfara State College of Education, Maru	Maru, Zamfara State.
**33**	Jigawa State College of Education, Gumel	Gumel, Jigawa State.
**34**	Niger State College of Education, Minna	Minna, Niger State.
**35**	Rivers College of Education, Rumuolumeni	Port-harcourt, Rivers State.
**36**	Shehu shagari College of Education, Sokoto	Sokoto, Sokoto State.
**37**	Jama’Atu College of Education (JACE), Kaduna	Kaduna, Kaduna State.
**38**	College of Education, Arochukwu, Abia	Arochukwu Abia State
**39**	College of Education, Ila-Orangun, Osun State	Ila-Orangun, Osun State
**40**	Michael Otedola Coll. of Prim. Education, Lagos	Noforija-Epe Lagos
**41**	Kashim Ibrahim College of Educ., Maiduguri	Borno State
**42**	Delta State Coll. of Physical Education, Mosogar	Mosogar, Sapele, Delta State
**43**	Enugu State Coll. of Education (T), Enugu	Abakaliki Road, Enugu State
**44**	Cross River State Coll. of Education, Akampa	Akampa, Calabar, Cross River State
**45**	Edo State College of Education, Igueben	Igueben, Edo State
**46**	Isaac Jasper Boro COE, Sagbama	Sagbama, Yenogoa, Bayelsa State
**47**	Kogi State College of Education, Kabba	Kabba, Kogi State

Table S11 | List of Private Colleges of Education in Nigeria (NBTE, 2021b; NCCE, 2021)

S/N	Names of Private Colleges of Education in Nigeria	Location
**1**	Institute of Ecumenical Education, (Thinkers Corner), Enugu	Enugu, Enugu State.
**2**	Delar College of Education	Agodi Gate, Ibadan, Oyo State
**3**	City College of Education, Mararaba, Gurku	Mararaba, Gurku, Nasarawa State
**4**	Ansar-Ud-Deen College of Education, Isolo	Oshodi, Isolo, Lagos State.
**5**	Yewa Central College of Education, Ayetoro	Ayetoro, Ogun State.
**6**	OSISA Tech. Coll. of Education, Enugu	Enugu, Enugu State
**7**	St. Augustine Coll. of Education, Lagos	Akoka, Lagos
**8**	African Thinkers Community of Inquiry, Enugu	Enugu, Enugu State
**9**	Muftau Olanihun College of Education, Ibadan	Ibadan
**10**	Havard Wilson College of Education, Aba	Aba, Abia State State
**11**	Muhyideen College of Education, Ilorin	Ilorin
**12**	College of Education, Offa	Offa, Kwara State
**13**	Bauchi Institute of Arabic & Islamic Studies, Bauchi	Bauchi
**14**	Corner Stone College of Education, lagos	Unilag, Lagos
**15**	Peaceland College of Education, Enugu	Enugu
**16**	The College of Education, Nsukka	Enugu
**17**	Unity College of Education, Auka Adoka, Benue	Otikpo
**18**	Diamond College of Education, Aba	Aba
**19**	Kinsey College of Education, Ilorin, Kwara State	Ilorin
**20**	ECWA College of Education, Jos (ECOEJ)	Jos

Table S12 | List of Federal Monotechnics in Nigeria (NBTE, 2021a)

S/N	Names of Federal Monotechnics in Nigeria	Location
1	Federal Cooperative College Ibadan	Oyo State.
2	Federal Cooperative College Kaduna	Kaduna State
3	Federal Cooperative College, Oji River	Enugu State.
4	Federal College of Statistics, Enugu	Enugu State
5	Federal College of Statistics, Ibadan	Oyo State
6	Federal College of Statistics, Kaduna	Kaduna State
7	Federal School of Mines, Jos	Plateau State
8	Federal School of Survey, Oyo	Oyo State
9	Federal Training Centre, Calabar	Cross River
10	Federal Training Centre, Enugu	Enugu State
11	Federal Training Centre, Kaduna	Kaduna State
12	Federal Training Centre, Maiduguri	Borno
13	Maritime Academy of Nigeria, Oron	Akwa Ibom State.
14	Metallurgical Training Institute, Onitsha	Anambra State.
15	National Water Resources Institute, Mando, Kaduna.	Kaduna State
16	Nigerian Army School of Engineering, Makurdi	Benue State.
17	Nigerian Army School of Finance, Administration, Apapa	Lagos State
18	Nigerian Army School of Signals Apapa, Lagos	Lagos State
19	Nigerian College of Aviation Technology (NCAT) Zaria	Kaduna State
20	Nigerian Naval Engineering College, Sapele,	Delta State
21	NITEL Training School, Oshodi,	Lagos State

Table S13 | List of State Monotechnics in Nigeria (NBTE, 2021c)

S/N	Names of State Monotechnics in Nigeria	Location
1	College of Administration and Business Studies, Konduga	Borno State.
2	College of Administration and Business Studies, Potiskum	Yobe State

Table S14 | List of Private Monotechnics in Nigeria (NBTE, 2021b)

S/N	List of Private Monotechnics in Nigeria	Location
1	Nigerian Institute of Journalism (NIJ)	Lagos State
2	Wavecrest College of Catering, Surulere	Lagos State

CONFLICT OF INTEREST

The authors declare that the research was conducted in the absence of any commercial or financial relationships that could be construed as a potential conflict of interest.

FUNDING

This research did not receive any specific grant from funding agencies in the public, commercial, or not-for-profit sectors.

REFERENCES

Commonwealth Network, CWN, (2021). Research Institutes in Nigeria. Retrieved January 27, 2021, from https://www.commonwealthofnations.org/sectors-nigeria/education/research_institutes/

Federal Ministry of Agricultural and Rural Development (FMARD), (2021). List of Research Institutes. Retrieved May 25, 2021 from https://fmard.gov.ng/reseach-institutes/

National Board for Technical Education, NBTE, (2021). Approved federal polytechnics. Retrieved January 11, 2021, from https://net.nbte.gov.ng/Federal%20Polytechnics

National Board for Technical Education, NBTE, (2021). Approved and accredited private polytechnics. Retrieved February 18, 2021, from https://net.nbte.gov.ng/Private%20Polytechnics

National Board for Technical Education, NBTE, (2021). Accredited state polytechnics. Retrieved February 23, 2021, from https://net.nbte.gov.ng/State%20Polytechnics

National Commission for Colleges of Education, NCCE, (2021). List of accredited colleges of education. Retrieved December 20, 2020 from https://www.ncceonline.edu.ng/colleges.php

National University Commission, NUC, (2021). Federal universities in nigeria. Retrieved April 14, 2021, from https://www.nuc.edu.ng/nigerian-univerisities/federal-univeristies/

National University Commission, NUC, (2021). Private universities in nigeria. Retrieved May 5, 2021, from https://www.nuc.edu.ng/nigerian-univerisities/private-univeristies/

National University Commission, NUC, (2021). State universities in nigeria. Retrieved May 10, 2021, from https://www.nuc.edu.ng/nigerian-univerisities/state-univerisity/

Nigerian Guide (2021). Full list of research institutes in nigeria. Retrieved May 22, 2021, from https://nigerianguide.com.ng/full-list-of-research-institutes-in-nigeria/

Copyright © 2021 Igiri, Okoduwa, Akabuogu, Okoduwa, Enang, Idowu, Abdullahi, Onukak, Onuruka, Christopher, Salawu, Chris and Onyemachi. This is an open-access article distributed under the terms of the Creative Commons Attribution License (CC BY). The use, distribution or reproduction in other forums is permitted, provided the original author(s) and the copyright owner(s) are credited and that the original publication in this journal is cited, in accordance with accepted academic practice. No use, distribution or reproduction is permitted which does not comply with these terms.
